# Comparison on cellular mechanisms of iron and cadmium accumulation in rice: prospects for cultivating Fe-rich but Cd-free rice

**DOI:** 10.1186/s12284-016-0112-7

**Published:** 2016-08-08

**Authors:** Lei Gao, Jiadong Chang, Ruijie Chen, Hubo Li, Hongfei Lu, Longxing Tao, Jie Xiong

**Affiliations:** 1 College of Life Sciences, Zhejiang Sci-Tech University, Hangzhou, 310018 People’s Republic of China; 2 Zhejinag Province Key Laboratory of Plant Secondary Metabolism and Regulation, Hangzhou, 310018 People’s Republic of China; 3 State Key Laboratory of Rice Biology, China National Rice Research Institute, Hangzhou, 310006 People’s Republic of China

**Keywords:** Iron, Cadmium, Biofortification, Mugineic acid, Phytosiderophores, Phytochelatins, Harvestplus, Fertilizer management

## Abstract

Iron (Fe) is essential for rice growth and humans consuming as their staple food but is often deficient because of insoluble Fe(III) in soil for rice growth and limited assimilation for human bodies, while cadmium (Cd) is non-essential and toxic for rice growth and humans if accumulating at high levels. Over-accumulated Cd can cause damage to human bodies. Selecting and breeding Fe-rich but Cd-free rice cultivars are ambitious, challenging and meaningful tasks for researchers. Although evidences show that the mechanisms of Fe/Cd uptake and accumulation in rice are common to some extent as a result of similar entry routes within rice, an increasing number of researchers have discovered distinct mechanisms between Fe/Cd uptake and accumulation in rice. This comprehensive review systematically elaborates and compares cellular mechanisms of Fe/Cd uptake and accumulation in rice, respectively. Mechanisms for maintaining Fe homeostasis and Cd detoxicification are also elucidated. Then, effects of different fertilizer management on Fe/Cd accumulation in rice are discussed. Finally, this review enumerates various approaches for reducing grain Cd accumulation and enhancing Fe content in rice. In summary, understanding of discrepant cellular mechanisms of Fe/Cd accumulation in rice provides guidance for cultivating Fe-fortified rice and has paved the way to develop rice that are tolerant to Cd stress, aiming at breeding Fe-rich but Cd-free rice.

## Review

Metal elements, such as Fe, Zn, Mn and Cu, are essential for living organisms and present as ions. Although there are abundant metal elements in the earth’s crust, these ions, particularly Fe, are sparingly soluble under aerobic conditions in high pH or calcareous soils and are not bioavailable to plants (Takahashi et al. 2003). As a result, Fe deficiency is a widespread agricultural problem that causes plants growth retardation and restricts sources of nutrition from plants (e.g., rice, maize and barley) (Mori 1999; Kobayashi et al. 2010). In response to Fe deficiency, higher plants have developed two strategies for acquiring Fe from the rhizosphere (Conte and Walker, 2011; Kobayashi and Nishizawa, 2012). The application of strategy I is non-graminaceous plants, which includes the reduction of Fe(III) to soluble Fe(II) by activating membrane-bound Fe(III)-chelate reductases, followed by uptake of the reduced Fe(II) into cytoplasm via Fe(II) transporters (Cheng et al. 2007). Strategy II is employed only by graminaceous plants, such as rice. Roots can secrete phytosiderophores (PSs) that belongs to the muginneic acid (MA) family to rhizosphere and chelate Fe(III), followed by uptake of Fe(III)-PS complexes via specific plasma membrane transporters (Conte and Walker, 2011). Rice utilizes strategy II to acquire Fe from rhizosphere and also possesses strategy I-like system that can take in Fe(II) directly (Cheng et al. 2007). In spite of rice can apply specific strategies to acquire Fe, these mechanisms have limited accessibility to resource-poor people faced with Fe deficiency from certain areas of the world. To deal with limited Fe and improve human Fe nutritional status, biofortifying rice with enhanced Fe absorption will be an effective method for populations consuming rice as their staple food.

Cd is a toxic heavy metal and accumulation of Cd in rice grains poses a latent health problem to human. Cd in human body can lead to chronic toxicity. The outbreak of “Itai-Itai disease” in the mid-20th century in Japan is due to consumption of Cd-contaminated rice (Uraguchi et al. 2011). A person with “Itai-Itai” has symptoms of weakness and softening of the bones (Horiguchi et al. 2010). Cd enters into environment, such as soil and river mainly through industrial activities or fertilizers (Bolan et al. 2003). As a mobile and soluble metal, Cd causes crops yield reduction and does harm to human health even at low concentrations (Choppala et al. 2014). The primary effects on plants caused by Cd-induced toxic symptoms are as follows: reduced rate of transpiration and photosynthesis, growth retardation and declining metabolic activities (Choppala et al. 2014). In response to Cd toxicity, plants have evolved protective mechanisms against Cd toxicity, including “avoidance” and “tolerance” (DalCorso et al. 2010). “Avoidance”, which means plants can prevent Cd from entering into cells and cell walls serve as the first parclose against Cd (Lang and Wernitznig, 2011; Choppala et al. 2014). Root exudates that majorly consist of sugars, proteins and organic acids are secreted from roots to soil, combining with Cd or keeping apart from roots (Schwab et al. 2005; Dong et al. 2007). After Cd inflows into cells, the abilities of resistance to Cd stress are referred to as “tolerance” (Choppala et al. 2014). A Cd chelator, phytochelatins (PC) plays a key role in Cd detoxification (Yadav et al. 2010). PC functions as chelating Cd in the cytosol and forming complexes with Cd. Complexes are sequestered in the vacuoles via specific transporters located at tonoplast (Ueno et al. 2010; Miyadate et al. 2011).

There is evidence that mechanisms of Fe/Cd uptake and accumulation in rice are common to some extent as a result of similar entry routes within rice. Nevertheless, an increasing number of researchers have discovered distinct mechanisms between Fe/Cd uptake and accumulation. This comprehensive review systematically elaborates and compares cellular mechanisms of Fe/Cd uptake and accumulation in rice at different stages, respectively. Mechanisms for maintaining Fe homeostasis and Cd detoxicification are elucidated. Effects of different fertilizer management on Fe/Cd accumulation in rice are discussed. Furthermore, this review enumerates various approaches for reducing grain Cd accumulation and enhancing Fe content in rice. In summary, understanding of discrepant cellular mechanisms of Fe/Cd accumulation in rice provides guidance for cultivating Fe-fortified rice and has paved the way to develop rice that are tolerant to Cd stress, aiming at breeding Fe-rich but Cd-free cultivars.

### Primary acquisition of Fe and Cd from rhizosphere to roots of rice

In strategy II of Fe acquisition, MAs are originated from *S*-adenosyl-l-L-methi-onine (SAM). SAM can be catalyzed by nicotianamine synthase (NAS) and produce nicotianamine (NA), which is an intermediate for the biosynthesis of MA family and a vital substance of nicotianamine aminotransferase (NAAT) (Kobayashi et al. 2010). Currently, three rice NAS genes, *OsNAS1*, *OsNAS2* and *OsNAS3* have been identified, playing different roles in Fe uptake and translocation (Inoue et al. 2003). NAAT is a critical enzyme in the biosynthesis of MAs that converts NA to 2’-deoxymugineic acid (DMA). Inoue et al. (2008) identified six rice *NAAT* genes (*OsNAAT1*-6), but only *OsNAAT1* was highly up-regulated under Fe deficiency, suggesting that *OsNAAT1*, but not *OsNAAT2-6*, encodes the sole functional enzyme possessing NAAT activity. DMA chelates Fe(III) and then forms Fe(III)-DMA complexes, which are absorbed by root cells (Cheng et al. 2007). Cheng et al. (2007) demonstrated that *NAAT1* mutant was not able to produce DMA and take up Fe(III) efficiently.

**Fig. 1:**
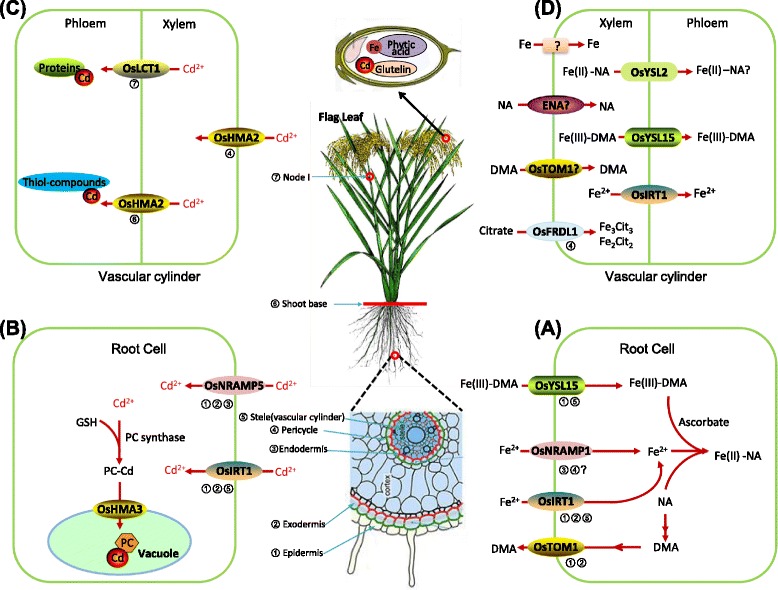
Mechanisms of Fe/Cd uptake and translocation in rice. **a** Fe uptake from rhizosphere into root cells by specific root transporters. DMA is synthesized in cells and secreted into the rhizosphere by OsTOM1. DMA chelates rhizospheric Fe(III), forming Fe(III)-DMA complexes. Complexes are then taken up into root cells by OsYLS15. Roots also take up Fe(II) directly by metal transporters (OsIRT1/OsNRAMP1). **b** Cd is absorbed from rhizosphere into root cells mediated by OsIRT1 and OsNRAMP5. OsHMA3 plays a critical role in Cd compartmentalization into vacuoles in root cells. **c** Cd xylem loading in roots for translocation to shoots by OsHMA2, and Cd phloem loading for storage to grain sink. OsLCT1 and OsHMA2 mediate xylem-to-phloem transfer at nodes. **d** Fe xylem loading in roots for translocation to shoots and the remobilization of Fe through phloem from leaves for storage to grain sink. OsFRDL1, which is a citrate transporter localized at the root pericycle cells. OsFRDL1 loads citrate into the xylem and combines with Fe. ENA may be involved in efflux of NA into xylem. OsYSL2 then mediate Fe(II)-NA for phloem loading. OsTOM1 potentially participates in DMA transport, followed by mediating Fe(III)-DMA through OsYSL15. Furthermore, OsIRT1 directly transports Fe(II) in phloem companion cells of shoots. The encircled numerals represent the main localization of specific transporters. Right parts of the figure are adapted partially from Kobayashi et al. (2014) and Yoneyama et al. (2015)

Under Fe-deficiency stress, transporters related genes for Fe uptake and translocation are transcriptionally induced (Kobayashi et al. 2014). As for rice, gene encoding DMA efflux transporters (OsTOM1) is highly expressed in response to low Fe availability (Nozoye et al. 2011). *OsTOM1* encodes TOM1 transporter that localizes at plasma membrane and mediates DMA secretion to rhizosphere, followed by Fe(III)-DMA complexes formation (Nozoye et al. 2011) (Fig. 1a). *Yellow stripe 1* (*YS1*) gene that encodes Fe(III)-MAs transporters was first acquired in maize. Maize *YS1* mutant presents interveinal chlorosis characteristic due to Fe deficiency (Curie et al. 2001). *YS1*-like (*OsYSL*) genes in rice have been subsequently identified over the decades, among which *OsYSL15* that transports Fe(III)-DMA is up-regulated in roots and shoots under Fe deficiency (Inoue et al. 2009). Fe(III)-DMA are absorbed via plasma membrane-bound OsYSL15 transporter (Inoue et al. 2009) (Fig. 1a). Furthermore, transporters that *OsYSL* genes encode are also involved in Fe translocation within rice (Koike et al. 2004; Kakei et al. 2012). Once inside the cytosol, Fe(III)-DMA can be reduced by ascorbate, forming Fe(II)-NA (Weber et al. 2008) (Fig. 1a). Hence, NA is not only an important intermediate for the biosynthesis of MAs, but also a significant metal chelator that can take part in translocation of Fe within plants (Takahashi et al. 2003).

In addition to Fe(III)-DMA uptake, rice also absorbs Fe (II) via iron-regulated transporter 1 (OsIRT1) and natural resistance-associated macrophage protein 1 (OsNRAMP1) under flooded conditions (Takahashi et al. 2011) (Fig. 1a). Seven rice *NRAMP* genes have been identified so far (Uraguchi and Fujiwara, 2012).

The recent research indicated that plasma membrane-localized protocatechuic acid (PCA) transporter, phenolic efflux zero1/2 (PEZ1/2), also participated in Fe uptake (Ishimaru et al. 2011). Such transporter played a role in absorbing apoplasmic precipitated Fe by secreting phenolics like PCA or caffeic acid. Suppression of *PEZ1/2* expression resulted in reduced Fe concentrations (Ishimaru et al. 2011; Kobayashi et al. 2014).

In comparison, Cd uptake from rhizosphere is a dose-dependent process and exhibits saturable kinetic characteristics in rice (Fujimaki et al. 2010; Ishikawa et al. 2011). Fujimaki et al*.* (2010) analyzed the kinetics of Cd uptake by roots in rice and suggested uptake rate of Cd was proportional to Cd concentration in the culture solution within a range from 0.05 to 100 nM, demonstrating a linear relationship between uptake rate and Cd concentration in a certain range. Ishikawa et al. (2011) suggested that this kinetic characteristic of Cd uptake could be mediated by transporters.

Cd enters into root cells via transporter OsNRAMP5 or OsIRT1 and OsNRAMP5 is predominantly applied (Nakanishi et al. 2006; Sasaki et al. 2012). *OsNRAMP5* expression is identified in roots epidermis, exodermis, and outer layers of the cortex as well as in tissues around the xylem (Ishimaru et al. 2012) (Fig. 1b). Knock-out of *OsNRAMP5* reduces Cd accumulation both in straw and grains slightly (Slamet-Loedin et al. 2015). Slamet-Loedin et al. (2015) also proposed that down-regulation of *OsNRAMP5* is a preferential strategy to decrease Cd uptake by roots. OsNRAMP5 not only mediates Cd uptake, but also manganese (Mn) uptake and relatively minor effect on Fe uptake under Fe starvation (Ishimaru et al. 2012) (Table 1). In addition, Takahashi et al. (2011) found that higher expression of *OsNRAMP1* in roots could enhance Cd accumulation in shoots of rice, indicating that OsNRAMP1 may take part in Cd uptake and transport besides Fe absorption (Takahashi et al. 2011). Consequently, such common characteristic of transporter-mediated acquisition mechanism paves the way for Cd accumulation in rice.

**Table 1:** Rice genes involved in Fe/Cd transport and induced status under Fe deficiency and Cd stress

Gene name	RAP ID	Function	Induced status under Fe deficiency and Cd stress^a^	References
NA/DMA biosynthesis for Fe(III)-DMA or Fe(II)-NA transport				
*OsNAS1*	Os03g0307300	Nicotianamine synthase	↑	Cheng et al. 2007
*OsNAS2*	Os03g0307200	Nicotianamine synthase	↑	Cheng et al. 2007
*OsNAS3*	Os07g0689600	Nicotianamine synthase	↑(root)↓(leaf)	Cheng et al. 2007
*OsNAAT1*	Os02g0306401	Nicotianamine aminotransferase	↑	Inoue et al. 2008
*OsDMAS1*	Os03g0237100	Deoxymugineic acid	↑	Kobayashi et al. 2014
Transporters for Fe/Cd uptake and translocation				
*OsTOM1*	Os11g0134900	DMA efflux transporter	↑	Nozoye et al. 2011
*OsYSL15*	Os02g0650300	Fe(III)-DMA transporter	↑	Inoue et al. 2009
*OsYSL16*	Os04g0542800	Fe(III)-DMA transporter	→	Kakei et al. 2012
*PEZ1*	Os03g0571900	Phenolics efflux transporter	―	Ishimaru et al. 2011
*PEZ2*	Os03g0572900	Phenolics efflux transporter	―	Ishimaru et al. 2012
*OsIRT1*	Os03g0667500	Ferrous Fe transporter	↑	Takahashi et al. 2011
*OsIRT2*	Os03g0667300	Ferrous Fe transporter	↑	Takahashi et al. 2011
*OsNRAMP1*	Os07g0258400	Ferrous Fe/Cd transporter	↑	Takahashi et al. 2011
*OsNRAMP5*	Os07g0257200	Ferrous Fe/Cd/Mn transporter	↑	Ishimaru et al. 2011
*OsFRDL1*	Os03g0216700	Citrate efflux transporter	→	Kobayashi et al. 2014
*ENA1*	Os11g0151500	NA efflux transporter	↑(root)↓(shoot)	Nozoye et al. 2011
*ENA2*	Os06g0695800	NA efflux transporter	↑(root)↓(shoot)	Nozoye et al. 2011
*OsYSL2*	Os02g0649900	Ferrous Fe/Mn-NA transporter	↑	Ishimaru et al. 2010
*OsHMA2*	Os06g0700700	Cd/Zn transporter	↑	Yoneyama et al. 2015
*OsLCT1*	Os06g0579200	Cd efflux transporter	―	Uraguchi et al. 2011
Transporters for cellular Fe/Cd sequestration				
*OsVIT1*	Os04g0463400	Fe import into vacuole	→	Pich et al. 2001; Zhang et al. 2012
*OsVIT2*	Os09g0396900	Fe import into vacuole	↓	Pich et al. 2001; Zhang et al. 2012
*OsHMA3*	Os07g0232900	Cd import into vacuole	―	Takahashi et al. 2012a
*OsABCG43*	Os07g0522500	Cd import into vacuole	―	Oda et al. 2011

^a^Arrows indicate rice genes expressional response: “↑”, upregulated; “↓”, downregulated; “→”, no significant change; “―”, not determined

After influx of Cd into cytosol, one significant pathway of Cd is sequestered into the vacuole via transporter OsHMA3 (Takahashi et al. 2012a) and transiently stored in the form of complexes (Choppala et al. 2014) (Fig. 1b). This pathway decreases Cd mobility in the cytosol and translocation from roots to shoots (Choppala et al. 2014; Shahid et al. 2016). *OsHMA3* is mainly expressed in roots (Miyadate et al. 2011). OsHMA3 belongs to P_1B_-ATPases and localizes at tonoplast (William and Mills, 2005). In *Arabidopsis thaliana*, AtHMA3, similar as OsHMA3, is responsible for sequestration of Cd into vacuoles (Miyadate et al. 2011). Meanwhile, an allele of *OsHMA3* was discovered to fail to transport Cd into vacuole in Cd-high-accumulating cultivars such as some *indica* cultivars. Owing to non-function of OsHMA3, Cd is accelerated to distribute within rice, leading to high accumulation. These cultivars presented high Cd accumulation in the shoots and grains (Miyadate et al. 2011).

### Translocation of Fe and Cd from roots to shoots

Following uptake by roots, Fe and Cd are transported to shoots via xylem and phloem, where exist a large amount of vascular bundles (Yoneyama et al. 2015). This radial transport system includes symplasmic and apoplasmic pathways, but the former pathway is predominantly utilized as a result of impediment by Casparian strips occuring in apoplasmic pathway (Enstone et al. 2002). After Fe(II)-NA formation in the cytosol, Fe(II)-NA is transported to xylem and exchanges NA with citrate, transforming to Fe(III)-citrate preferentially (Yokosho et al. 2009; Ariga et al. 2014). Fe in the xylem is largely in the form of Fe-citrate and then allocated to all leaves, whereas Fe in the phloem is mainly bound to DMA, citrate and proteins (Yoneyama et al. 2015). The translocation of citrate from root pericycle cells to xylem is mediated by ferric reductase defective1-like transporter (OsFRDL1) (Yokosho et al. 2009) (Fig. 1d). *OsFRDL1* is constitutively expressed in root pericycle cells and transporter OsFRDL1 is specifically required for Fe translocation (Yokosho et al. 2009).

Phloem loading is the upcoming step. Transporter OsYSL2 plays a part in Fe distribution in the phloem, localizing at the plasma membrane and is responsible for Fe(II)-NA or Mn(II)-NA transport, but not for Fe(III)-DMA transport (Ishimaru et al. 2010) (Table 1). OsYSL2 knock-down rice lines accumulated less Fe and Mn in shoots and seeds (Kobayashi et al. 2010). With regard to mechanism for efflux of NA into xylem in specific way, Nozoye et al. (2011) proposed that the NA efflux transporters (ENA1/2) are responsible for the effux of NA into xylem or intracellular compartments in order to redistribute Fe (Fig. 1d). Under Fe deficiency, both *OsYSL2* and *ENA1* are strongly induced (Ishimaru et al. 2010; Ogo et al. 2014). In addition to transporter OsYSL2, OsYSL15 is considered to transport Fe(III)-DMA for phloem trafficking and expressed in the phloem companion cells (kobayashi et al. 2010; kakei et al. 2012; Kobayashi et al. 2014) (Fig. 1d). Thereafter, Fe is delivered to grain via phloem in forms of Fe(III)-DMA or binds to some citrate and proteins (Yoneyama et al. 2015).

As for Cd translocation, once Cd enters into root cells, part of Cd present as Cd-phytochelatin (Cd-PC) complexes are sequestered in the vacuoles and the others are transported to xylem mediated by OsHMA2 transporter. Such xylem loading occurs in root pericycle cells with OsHMA2 (Takahashi et al. 2012b; Yamagi et al. 2013; Yoneyama et al. 2015) (Fig. 1c). Moreover, OsHMA2 is also involved in xylem-to-phloem transfer (Yoneyama et al. 2015). In the phloem, Cd primarily bounds to specific proteins and slightly to thiol-compouds (White and Broadley 2011). In contrast to Fe translocation that is mainly derived from leaves by remobilization, xylem-to-phloem transfer system of Cd mainly occurs at the nodes (Fujimaki et al. 2010). In rice nodes, the diffuse vascular bundles (DVBs) that encircle the enlarged elliptical vascular bundles (EVBs) are connected to the panicle (Yamaguchi et al. 2012). A study demonstrated that Cd was predominantly transported towards the panicle instead of other tissues at the panicle-initiation stage through the nodes and ultimately reached grain by prositron-emitting ^107^Cd tracer imaging system (PETIS) (Fujimaki et al. 2010). Node I, the uppermost node, is connected to both flag leaf and panicle. The large vascular bundles (LVBs) of flag leaf are linked to the EVBs. Metals, such as Cd, that are not transported to the panicle can be shifted to flag leaf (Uraguchi et al. 2011). Yamaguchi et al. (2012) found that Cd concentration was higher in the node I than in blade, culm and panicle due to the accumulation of Cd. Furthermore, A low-affinity cation transporter (OsLCT1), which is highly expressed in the node I, participates in Cd transport to grain (Uraguchi et al. 2011) (Fig. 1c). OsLCT1 is identified as a plasma membrane-localized transporter by subcellular localization of OsLCT1-sGFP (Uraguchi et al. 2011). Suppression of *OsLCT1* exppression can efficiently decrease grain Cd levels (Uraguchi et al. 2011).

Therefore, Node is deemed to be an important “transportation junction” responsible for Cd distribution. Shoot base contains the lower packed nodes with numerous vascular bundles, designated as the “traffic control centre”, and where Cd can be separated into the tillers, accumulating in each node (Fujimaki et al. 2010).

### Role of cellular sequestration in mitigating Cd toxicity

As stated in the introduction section above, Plants have developed numerous resistance mechanisms against Cd toxicity, “avoidance” and “tolerance” included. “Avoidance” is first employed and serves as a basic mechanism to relieve Cd toxicity. However, “tolerance”, which makes plants survive in the presence of Cd stress, plays a major role in mitigating Cd toxicity. In the “tolerance” mechanisms, deposition of Cd in the cell wall is a first barrier to restrict Cd movement by combining with composition of cell wall (Carrier et al. 2003; Choppala et al. 2014) (Table 2). Xiong et al. (2009) first proved that exogenous NO could be involved in regulation of root cell wall composition to alleviate Cd toxicity. Enhanced pectin and hemicellulose contents (Table 2) induced by exogenous NO in root cell wall increased Cd deposition in cell wall and decreased Cd distribution within rice. Once entering into cell, PC that acts as a chelator can make complexes with Cd, forming Cd-PC (Table 2). Basic structure of PC, consisting of glutamate, cysteine and glycine has been identified (Rauser 1995), and glutathione (GSH) is a key intermediate for the biosynthesis of PC and catalyzed by PC synthase (PCS) (Rauser 1995; Cobbett 2000). PCS can be activated in the presence of Cd (Cobbett 2000). As for rice, in the root cells, Cd-PC complexes are stored in the vacuoles mediated by tonoplast-localized transporter OsHMA3 (Tables 1 and 2), followed by dissociation in the vacuoles due to acidic environment (Johanning and Strasdeit 1998; Takahashi et al. 2012a, b; Choppala et al. 2014). Dissociated PC can be recycled in the next round (Johanning and Strasdeit 1998).

**Table 2:** Comparison on mechanisms of Fe/Cd detoxicfication in rice

Mechanisms	Fe	Cd	References
Cellular sequestration			
Storage sites	Vacuole in the flag leaves and sheaths	Vacuole in the root cells and leaves	Choppala et al. 2014; Pich et al. 2001;
Chemical forms	Fe(II)-NA	Cd-PC	Takahashi et al. 2012a;
Mediated transporters	OsVIT1/2	OsHMA3	Zhang et al. 2012
Location of transporters	Tonoplast	Tonoplast	
Combination of Fe/Cd and organics			
Storage sites	Chloroplast or embryo in the leaves and seeds	Cell wall in the roots and leaves	Ravet et al. 2009; Xiong et al. 2009
Chemical forms	Fe-Ferritin	Cd-Pectin and Cd-Hemicellulose	

According to this cellular mechanism, subsequent movement of Cd through the root symplasm to the xylem can be limited (Nocito et al. 2011). Some reports pointed that OsHMA3 is expressed in shoots besides roots, which implies the key factor of OsHMA3 in determining root-to-shoot transfer of Cd and regulation of Cd distribution within rice. Particularly, cellular sequestration mediated by OsHMA3 in root cells is a rate-limiting step (Ueno et al. 2010). Furthermore, an ABC-type transporter OsABCG43 is also considered to be a probable candidate for Cd tolerance in rice (Oda et al. 2011; Uraguchi and Fujiwara 2012). OsABCG43 is likely to sequester Cd at the subcellular level, as well as vacuolar sequestration mediated by OsHMA3 (Oda et al. 2011) (Table 1). Nevertheless, whether there are other mechanisms mediated by OsABCG43 is far from clear.

### Mechanisms for maintaining Fe homeostasis

Despite enhancing Fe is primary concern to agricultural production, over-accumulated Fe can cause cellular damage since Fe is highly reactive (Conte and Walker, 2011). Fe(II) is considered to be a source of reactive oxygen species (ROS), which results in oxidative damage (Curie et al. 2009). Thus, maintaining Fe homeostasis is crucial to plant growth. Plant has evolved Fe homeostatic mechanisms that regulate Fe acquisition. Once taken up into cells, Fe is subjected to strict control to avoid cellular damage. Ferritin, a Fe storage protein, can combine with Fe atoms in bioavailable and non-toxic form for distribution within plants and protect against Fe-mediated oxidative stress (Liu et al. 2003; Ravet et al. 2009) (Table 2). Besides ferritin, nitric oxide (NO) is a promising candidate for serving as a scavenger of ROS, and the reduction of nitrite by nitrite reductase (NR) is a major pathway to generate NO (Crawford 2006). Moreover, vacuolar sequestration is another significant mechanism in controlling Fe homeostasis. Vacuole functions as buffering pool in conditions of Fe toxicity through the interaction between tonoplast-localized transporters and Fe chelators (Table 2). As mentioned from the above, NA is also involved in intracellular movements and acts as a strong chelator of Fe (Table 2). Excess Fe, as well as Cd compartmentalization, can be chelated by NA and sequestrated in the vacuole (Pich et al. 2001). In rice, *OsVIT1* and *OsVIT2* that encode vacuolar transporters are highly expressed in flag leaf and sheath, respectively, transporting excess Fe into vacuole (Zhang et al. 2012) (Tables 1 and 2).

### Effects of fertilizers on Fe and Cd accumulation discrepancy in rice

Enhancing Fe concentration in grains through water-fertilizer management is a kind of agronomic biofortification though the strategy plays a role in a short term (Slamet-Loedin et al. 2015). Nitrogen (N) is an essential macronutrient for plants (Sarwar et al. 2010). N application promotes YSL protein synthesis and nitrogenous compounds formation, such as NA and DMA, both of which participate in Fe transport in rice (Slamet-Loedin et al. 2015) (Table 3). Moreover, increased N application results in more biomass production and reduces Cd toxicity to some extent due to dilution effect (Sarwar et al. 2010). Such effect is mainly caused by increased soluble protein content in crops that can transform mobile Cd to immobile form by binding (Sarwar et al. 2010) (Table 3). Different N fertilizer forms also have relationships with Fe/Cd uptake and accumulation in both roots and shoots (Mitchell et al. 2000). Ammonium (NH_4_^+^) and nitrate (NO_3_^−^) are primary N fertilizer forms for rice absorption and assimilation (Jalloh et al. 2009). In the paddy soil, NH_4_^+^-containing fertilizer is predominantly employed (Araki et al. 2015). Higher antioxidase activity exposure to NH_4_^+^ treatment is considered as protective mechanism against Cd stress (Rizwan et al. 2016) (Table 3). Although the preferential application of NH_4_^+^ over NO_3_^−^ as a nitrogen source for rice, many reports proved that combination of NH_4_^+^ and NO_3_^−^ is better for rice growth (Sarwar et al. 2010; Araki et al. 2015).

**Table 3:** Positive and negative effects caused by fertilizer types on Fe/Cd accumulation in rice

Fertilizer types	Positive effects	Negative effects	References
Nitrogen (NH^4+^/NO^3−^)	Increased YSL protein synthesis and nitrogenous compounds formation for Fe transport	Decreased soil pH and membrane depolarization by NH^4+^ application	Zaccheo et al. 2006; Wangstrand et al. 2007; Xie et al. 2009; Sarwar et al. 2010; Slamet-Loedin et al. 2015; Yang et al. 2016b
	Increased soluble protein content reduce mobility of Cd	Up-regulated expression of Fe/Cd co-transporters by excess NO^3−^ application	
	Higher antioxidase activity by NH^4+^ application reduce Cd toxicity		
	High soil pH and membrane polarization by NO^3−^ application produce Cd detoxcification		
Phosphorus	Insoluble Cd formation in soil	Limited source of P fertilizer	Cordell et al. 2009; Wang et al. 2009; Sarwar et al. 2010
	GSH biosynthesis participation	Decreased soil pH enhance solubility of Cd	
	Increased antioxidase activity by P application		
Iron	Compete with Cd for the same binding site under anaerobic conditions	Increased Cd concentration by some Fe^2+^ fertilizers (e.g. FeSO_4_) application	Sharma et al. 2004; Shao et al. 2008; Liu et al. 2008; Rizwan et al. 2016
	Alleviate oxidative stress caused by Cd		
	Iron plague formation		
Zinc	Compete with Cd for the same transporters	Simultaneous Zn/Cd absorption by root cells	Smilde et al. 1992; Aravind et al. 2009; Sarwar et al. 2010; Fahad et al. 2015; Rizwan et al. 2016
	Alleviate oxidative stress caused by Cd	Enhanced Cd concentration caused by high level of Zn	
Silicon	Increased soil pH reduce mobility of Cd		Sarwar et al. 2010; Wang et al. 2015; Rizwan et al. 2016
	Si-Cd complexes formation		
	Enhanced antioxidase activity		
	Enhanced Fe level		
Sulfur	Insoluble CdS formation reduce mobility of Cd	Increased soil pH enhance Cd concentration and mobility	Hassan et al. 2005; Rehman et al. 2015
	GSH biosynthesis participation		
	Iron plague formation		

Cd in acidic soil is ionized as Cd^+^ (Khaokaew et al. 2011), which can promote mobilization of Cd (Sarwar et al. 2010). Rhizosphere acidification caused by NH_4_^+^-containing fertilizer derives from proton excretion by root cells, exchanging with NH_4_^+^ and leading to low pH in soil (Zaccheo et al. 2006). In low pH soil, Cd is mobile to move towards root system and translocates within plants, resulting in Cd accumulation (Table 3). In addition, NH_4_^+^ can trigger cell membrane depolarization and lead to influx of NH_4_^+^ into root cells, which accelerates translocation of Cd from root to shoot though this mechanisms reduces Cd uptake in a certain way (Zaccheo et al. 2006; Sarwar et al. 2010) (Table 3). Consequently, NH_4_^+^-containing fertilizer is considered to contribute to enhance Cd uptake (Sarwar et al. 2010). Compared with NH_4_^+^-containingfertilizer, NO_3_^−^-containing fertilizer causes simultaneous NO_3_^−^ and proton absorption by root cells, leading to high pH (Eriksson, 1990) and cell membrane polarization caused by nitrate can produce Cd detoxification mechanism (Sarwar et al. 2010) (Table 3). Nevertheless, Xie et al. (2009) found that plants supplied with NO_3_^−^ accumulated more Cd than NH_4_^+^ treatment by *Thlaspi caerulesscens* in hydroponic experiment, suggesting that effects of NH_4_^+^ and NO_3_^−^ on Cd uptake are not simply attributed to rhizosphere pH transformation or charge distribution of cell membrane. Yang et al. (2016b) found that rice fed with excess NO_3_^−^ not only enhanced Fe uptake, but also increased Cd uptake by up-regulating the expression of *OsIRT1.* It deserves further study that N fertilizer might enhance Cd uptake and accumulation resulting from up-regulated expression of common Cd/Fe transporter genes (Table 3). Wangstrand et al. (2007) once proposed that application of N fertilizer is dependent on different growth stages and recommended that more N fertilizer should be applied at the vegetative stage while less N doses should be applied during the grain filling stage. Therefore, the application of N fertilizer on rice can be manipulated to decrease Cd content and enhance Fe content. Meanwhile, selection of proper N fertilizer forms according to different growth stages is still hot research field.

Besides application of N fertilizer, other mineral fertilizers also contribute to rice growth as well. Phosphorus (P) is another important plant nutrient and applied to plant as fertilizer. In contrast to N, P is a non-renewable natural resource and there is growing concern about limited source of P fertilizer (Cordell et al. 2009). Most of P are derived from rock phosphate containing Cd and hence result in Cd contamination in soil (Lambert et al. 2007; Sarwar et al. 2010). Sarwar et al. (2010) mentioned that mono-ammonium-phosphate (MAP) could enhance Cd uptake due to increased solubility of Cd by lowering soil pH (Table 3). However, P fertilizer also reduce solubility of Cd by insoluble Cd formation, such as Cd(OH)_2_ or Cd_3_(PO_4_)_2_ (Bolan et al. 2003) (Table 3). Furthermore, crops have developed intrinsic mechanisms against Cd stress caused by rock phosphate containing Cd (Sarwar et al. 2010; Slamet-Loedin et al. 2015). Slamet-Loedin et al. (2015) mentioned that minor effect of P fertilizer-related Cd uptake on rice. P is involved in glutathione (GSH) biosynthesis (Table 3), which is precursor of PC. Recently, Yang et al. (2016a) proposed that P deprivation decreases Cd uptake by inhibiting biomass accumulation and reducing PCs synthesis. As elucidated above, in rice, PC can form complexes with Cd after Cd is transported into vacuole, alleviating Cd toxicity efficiently (May et al. 1998; Sarwar et al. 2010). On the other hand, an increase in antioxidase activity by the application of P plays an indispensable role in alleviating oxidative stress caused by Cd toxicity (Wang et al. 2009) (Table 3). In summary, as well as N application, appropriate P application is necessary for rice growth.

Application of Fe fertilizer is direct and effective method for enhancing Fe content while reducing Cd toxicity to some extent. Under anaerobic conditions, such as flooded status, Fe^2+^ is dominant chemical form in soil (Sarwar et al. 2010). Owing to similar chemical form, Fe^2+^ compete with Cd for the same binding site and transport systems on the surface of root cells (Table 3), reducing Cd uptake in a certain way accordingly (Sarwar et al. 2010). Nevertheless, different types of Fe^2+^ fertilizer may have discrepant effects on Cd accumulation. Shao et al. (2008) showed that application of FeSO_4_ remarkably increased Cd concentrations in roots and shoots of rice (Table 3). Thus, selection of appropriate Fe type is conducive to rice growth. Furthermore, Fe is important co-factor of antioxidase, which can provide protective mechanisms against oxidative stress (Sharma et al. 2004) (Table 3). A peculiar mechanism against Cd stress by application of Fe fertilizer is iron plague (IP) formation (Rizwan et al. 2016) (Table 3). This IP can serve as a barrier and prevent Cd from entering into root cells, resulting in reduced Cd accumulation while enhanced Fe concentration in rice (Liu et al. 2008).

Zinc is an essential micronutrient for crops growth. As a result of similar physical and chemical characteristics (Rizwan et al. 2016), the interactions between Zn and Cd, synergistic and antagonistic effect (Fahad et al. 2015; Rizwan et al. 2016), have been an issue to be solved for the field of agricultural science. In antagonistic way, Zn will compete with Cd for the same membrane transporters (Table 3), restricting Cd uptake by root cells. In synergistic way, both of Zn and Cd are absorbed by root cells, increasing Cd accumulation in rice (Sarwar et al. 2010) (Table 3). Xue and Harrison (1991) discovered that higher level of Zn fertilizer application causes enhanced Cd concentration in lettuce leaves. Smilde et al. (1992) also identified that increased Zn concentration in soil leaded to high Cd concentration in leafy vegetables (Table 3). Hence, controlling Zn fertilizer application at an optimum level may reduce Cd concentration caused by synergistic effect. In addition, Zn application is involved in alleviating oxidative stress (Table 3) caused by Cd and has protective mechanisms against reactive oxygen species (ROS) that result in cell membrane damage (Aravind et al. 2009).

Silicon (Si) is thought to be an enhancer for crops growth though it is not an essential element (Rizwan et al. 2016). Wang et al. (2015) found that Si application could enhance Fe level in rice (Table 3). Si application can reduce mobility of Cd due to increased pH in soil (Sarwar et al. 2010) (Table 3). Complexes formation of Si with Cd is another mechanism for alleviating Cd toxicity in rice (Rizwan et al. 2016) (Table 3). Moreover, Si application can also enhance antioxidase activity (Sarwar et al. 2010) (Table 3).

Sulfur is a significant component of many co-factor of enzymes (Table 3), having an indispensable effect on crops growth (Sarwar et al. 2010). Application of S fertilizer may decrease Cd toxicity by insoluble CdS formation (Table 3), by which reduces mobility of Cd in soil (Hassan et al. 2005). As well as effect of P fertilizer application, S also participates in GSH biosynthesis (Table 3), forming Cd-PC complexes and reducing Cd toxicity by compartmentalization of Cd into vacuole (Cobbett 2000; Sarwar et al. 2010; Rizwan et al. 2016). However, Rehman et al. (2015) suggested that application of S fertilizer might enhance Cd concentration in rice grains by lowering soil pH and increase mobility of Cd (Table 3), which is negative effect of S fertilizer application. Therefore, Rizwan et al. (2016) proposed that combination of different mineral fertilizers contributed to decrease Cd uptake by rice compared with respective application. For instance, Wei et al. (2012) found that foliar application of combined Fe and Zn fertilizers might increase Fe and Zn content, decreasing Cd content in rice grains.

### Approaches for reducing grain Cd accumulation and enhancing Fe content

Soil remediation methods are traditionally applied to reduce Cd toxicity to some extent, including soil removal, replacement, inversion and flooded condition before and after heading (Arao et al. 2009; Uraguchi and Fujiwara 2012). In addition, establishment of “low-Cd-rice” based on genetic findings is considered to be an effective approach to reduce Cd accumulation. There is genotypic variation in the Cd levels of grains in different rice cultivars. Cd accumulation in shoots and grains are greater in *indica* rice cultivars than in *japonica* cultivars (Ishikawa et al. 2005; Takahashi et al. 2011). Quantitative trait locus (QTL) analysis is a useful approach to identify responsible genes for the respective transport processes, such as various transporters (Ishikawa et al. 2010). QTL for Cd concentration in Anjana Dhan (*indica* rice cultivar) is identified on chromosome 7, responsive gene for which is *OsHMA3* (Ishikawa et al. 2010; Ueno et al. 2010; Takahashi et al. 2011). Amino acid at position 80 plays a key role in the function of OsHMA3 and mutation of this amino acid in Anjana Dhan makes Cd fail to be sequestered into vacuoles in root cells, accelerating translocation of Cd from roots to shoots (Ueno et al. 2010; Takahashi et al. 2011). Abe et al. (2011) introduced a non-functional allele of *OsHMA3* from Jarian (*indica* rice cultivar) into Koshihikari (*japonica* rice cultivar) by marker-assisted selection and these plants showed reduced Cd uptake from soil. Regulation of genes for Cd transporters can also effectively reduce Cd accumulation in rice (Ueno et al. 2010). Suppression of *OsLCT1* expression can decrease grain Cd accumulation by RNAi without influencing nutrient accumulation. On the contrary, Fe content in the brown rice is remarkably higher (Uraguchi et al. 2011), suggesting that RNAi-mediated *OsLCT1* suppression in rice is a promising approach to establish “high Fe but low-Cd-rice”. Furthermore, a novel rice gene *low cadmium* (*LCD*) is related to Cd tolerance (Uraguchi and Fujiwara, 2012). This T-DNA-mediated *OsLCD* knockout mutant showed reduced grain Cd accumulation by insertion into the first intron of *OsLCD*, having no negative effects on grain yield (Shimo et al. 2011). The authors indicated that the *lcd* mutant might be a probable mutant line for further research.

Improving bioavailability of Fe is the main goal for breeding Fe-rich rice. A variety of approaches have been utilized to enhance Fe content in grains. Goto et al. (1999) demonstrated that high level of Fe in rice endosperm could be acquired by overexpression of ferritin. Combination of up-regulated expression of ferritin with overproduction of NA can significantly enhance Fe content (Wirth et al. 2009). Zheng et al. (2010) indicated that biofortifying rice with NA could efficiently enhance Fe bioavailability by over-expression *OsNAS1* in rice endosperm, suggesting that NA plays a great potential role in enhancing Fe bioavailability. In addition, manipulation of specific transporters involved in Fe uptake and translocation is considered to be another promising approach for enhancing Fe content. Ishimaru et al. (2010) introduced *OsYSL2* mediated by sucrose transporter (OsSUT1) promoter into rice plants due to location of OsSUT1 around endosperm, resulting in high concentration of Fe in polished rice.

In recent years, studies on rice screened for Fe-rich but Cd-free cultivars have been an important issue to agricultural field. Olive et al. (2014) bred an over-accumulated ferritin cultivars with rice mega-variety IR64 that serve as background. Compared with IR64 wild type, Fe content was increased in grains by introducing ferritin into endosperm. Such a ferritin-bioengineered diet is considered to be effective for mammal assimilation of Fe nutrition (Murray-Kolb et al. 2002; Olive et al. 2014). Moreover, Booyaves et al. (2016) expressed Arabibopsis IRT1(*AtIRT1*) in high-iron NFP rice lines, which expressed *NICOTIANAMINE SYNTHASE* (*AtNAS1*) and *FERRITIN*, suggesting that coordinated expression of *AtIRT1*, *AtNAS1* and *PvFERRITIN* enhanced Fe content in both unpolished and polished grains. Thus, combined expression of genes encoding Fe transporters or Fe storage proteins are promising approach for Fe biofortification. With regard to breeding low Cd cultivars, Ishikawa et al. (2012) identified and screened three low-Cd mutants (*lcd-kmt1*, *lcd-kmt2* and *lcd-kmt3*) with *japonica* rice cultivar, Koshihikari, which acted as parent by the way of carbon ion-beam irradiation, showing that there were lower Cd concentration in grains of the three mutants than Koshihikari wide type (WT). Such three low-Cd mutants were attributed to mutations of *OsNRAMP5* responsible for Cd transport in rice by sequence analysis (Ishikawa et al. 2012). The three low-Cd mutants have different mutation sites in *OsNRAMP5*. An insertion of transposon *mPingA1*, which was activated by ion beam and preferred to insert into exon of *OsNRAMP5* was identified in *lcd-kmt1*, resulting in non-function of OsNRAMP5 and decreased Cd accumulation in grains (Ishikawa et al. 2012). Similar results were observed in *lcd-kmt2* and *lcd-kmt3* due to a single-base pair deletion and a large deletion in *OsNRAMP5*, respectively (Ishikawa et al. 2012). Meanwhile, Ishikawa et al. (2012) proposed that *lcd-kmt1* and *lcd-kmt2* were more promising for breeding program according to agronomic traits, as a consequence of earlier heading and smaller plant size than Koshihikari WT in *lcd-kmt3* (Ishikawa et al. 2012). In addition, Abe et al. (2013) developed a novel population composed of 46 chromosome segment substitution lines (CSSLs), in which LAC23 served as donor segments and were substituted into background, Koshihikari. LAC23 could result in lower grain-to-straw ratio than Koshihikari (Abe et al. 2013). Therefore, Cultivars containing LAC23 performed low Cd content in grains (Abe et al. 2013). QTL mapping detected a major QTL, *qlGCd3*, and LAC23 allele at *qlGCd3* were identified to be related with reduced Cd content in grains (Abe et al. 2013). This result showed that low-Cd trait of LAC23 is promising for breeding low-Cd rice cultivars.

## Conclusions and perspectives

Fe is essential nutrient for rice growth and humans consuming rice as their staple food, whereas Cd is non-essential and toxic. Rice grains contain both Fe and Cd. The latter may cause damage to human bodies if accumulating at high levels. Basic transport processes are as follows: During the vegetative stage, Fe and Cd are absorbed by specific root transporters and then transport to xylem, delivering to aerial parts via xylem-to-phloem transfer system. Compared with Cd of which such transfer system mainly operating at nodes, Fe is preferentially allocated to leaves through xylem. At grain-filling, grain Fe and Cd are derived from phloem. Particularly, grain Fe is largely acquired from leaves by remobilization. Due to limited source of Fe in soil and Cd toxicity, rice has evolved mechanisms against Fe deficiency and Cd stress. Rice can secrete DMA to chelate insoluble Fe^3+^ by strategy II. In response to Cd toxicity, compartmentalization of Cd into vacuole is thought to be effective tolerance mechanism for reducing Cd translocation to grains. However, relying on rice self-mechanisms are not enough to tackle issues of Fe deficiency and Cd toxicity. Besides traditional soil remediation and fertilizers management, breeding high Fe but low Cd cultivars through genetic methods are considered as promising approaches based on understanding of Fe/Cd transport and accumulation processes at cellular level. QTL analysis, regulation and manipulation of genes involved in Fe/Cd accumulation are generally utilized to produce novel cultivars.

Despite these advances in enhancing Fe in grains, limited Fe in edible parts due to combining with phytic acid and inevitable loss during polishing process restricts Fe nutritional assimilation for human bodies. In addition, some low-Cd cultivars are reported to enhance other toxic metals, such as Arsenic, though reduce Cd content to some extent. Thus, further investigation into these issues will be conducive to develop Fe-fortified cultivars with increased assimilation and low-Cd cultivars without other toxic metals accumulation.

## References

[CR1] Abe T, Taguchi-Shiobara F, Kojima Y, Ebitani T, Kuramata M, Yamamoto T, Yano M, Ishikawa S (2011). Detection of a QTL for accumulating Cd in rice that enables efficient Cd phytoextraction from soil. Breeding Sci.

[CR2] Abe T, Nonoue Y, Ono N, Omoteno M, Kuramata M, Fukuoka S, Yamamoto T, Yano M, Ishikawa S (2013). Detection of QTLs to reduce cadmium content in rice grains using LAC23/ Koshihikari chromosome segment substitution lines. Breeding Sci.

[CR3] Araki R, Kousaka K, Namba K, Murata Y, Murata J (2015). 2′-Deoxymugineic acid promotes growth of rice (*Oryza sativa*) by orchestrating iron and nitrate uptake processes under high pH conditions. Plant J.

[CR4] Arao T, Kawasaki A, Baba K, Mori S, Matsumoto S (2009). Effects of water management on cadmium and arsenic accumulation and dimethylarsinic acid concentrations in Japanese rice. Environ Sci Tech.

[CR5] Aravind P, Prasad MN, Malec P, Waloszek A, Strzalka K (2009). Zinc protects *Ceratophyllum demersum* L. (free-foating hydrophyte) against reactive oxygen species induced by cadmium. J Trace Elem Med Biol.

[CR6] Ariga T, Hazama K, Yanagisawa S, Yoneyama T (2014). Chemical forms of iron in xylem sap from graminaceous and non-graminaceous plants. Soil Sci Plant Nutr.

[CR7] Bolan N, Adriano DC, Duraisamy P, Mani A, Arulmozhiselvan K (2003). Immobilization and phytoavailability of cadmium in variable charge soils. I. Effect of phosphate addition. Plant Soil.

[CR8] Booyaves K, Gruissem W, Bhullar NK (2016). *NOD* promoter-controlled *AtIRT1* expression functions synergistically with *NAS* and *FERRITIN* genes to increase iron in rice grains. Plant Mol Biol.

[CR9] Carrier P, Baryla A, Havaux M (2003). Cadmium distribution and microlocalization in oilseed rape (*Brassica napus)* after long-term growth on cadmium-contaminated soil. Planta.

[CR10] Cheng LJ, Wang F, Shou HX, Huang FL, Zheng LQ, He F, Li JH, Zhao FJ, Uneo D, Ma JF, Wu P (2007). Mutation in nicotianamine aminotransferase stimulated the Fe(II) acquisition system and led to iron accumulation in rice. Plant Physiol.

[CR11] Choppala G, Saifullah Bolan N, Bibi S, Iqbal M, Rengel Z, Kunhikrishinan A, Ashwath N, Ok YS (2014). Cellular mechanisms in higher plants governing tolerance to cadmium toxicity. Crit Rev Plant Sci.

[CR12] Cobbett CS (2000). Phytochelatins and their roles in heavy metal detoxicification. Plant Physiol.

[CR13] Conte SS, Walker EL (2011). Transporters contributing to iron trafficking in plants. Mol Plant.

[CR14] Cordell D, Drangert JO, White S (2009). The story of phosphorus: Global food security and food for thought. Global Environ Change.

[CR15] Crawford NM (2006). Mechanisms for nitric oxide synthesis in plants. J. Exp Bot.

[CR16] Curie C, Panaviene Z, Loulergue C, Dellaporta SL, Brait JF, Walker EL (2001). Maize *yellow stripe1* encodes a membrane protein directly involved in Fe(III) uptake. Nature.

[CR17] Curie C, Cassin G, Couch D, Divol F, Higuchi K, Le Jean M, Misson J, Schikora A, Czernic P, Mari S (2009). Metal movement within the plant: contribution of nicotianmine and yellow stripe1-like transporters. Ann Bot.

[CR18] DalCorso G, Farinati S, Furini A (2010). Regulatory networks of cadmium stress in plants. Plant Signal Behav.

[CR19] Dong J, Mao WH, Zhang GP, Wu FB, Cai Y (2007). Root excretion and plant tolerance to cadmium toxicity-a review. Plant Soil Environ.

[CR20] Enstone DE, Peterson CA, Ma F (2002). Root endodermis and exodermis: structure, function, and responses to the environment. J Plant Growth Regul.

[CR21] Eriksson JE (1990). Effects of nitrogen-containing fertilizers on solubility and plant uptake of cadmium. Water Air Soil Pollut.

[CR22] Fahad S, Hussain S, Khan F, Wu C, Saud S, Hassan S, Ahmad N, Gang D, Ullah A, Huang J (2015). Effects of tire rubber ash and zinc sulfate on crop productivity and cadmium accumulation in five rice cultivars under field conditions. Environ Sci Pollut Res Int.

[CR23] Fujimaki S, Suzui N, Ishioka NS, Kawachi N, Ito S, Chino M, Nakamura S (2010). Tracing cadmium from culture to spikelet: Noninvasive imaging and quantative characterization of absorption, transport, and accumulation of cadmium in an intact rice plant. Plant Physiol.

[CR24] Goto F, Yoshihara T, Shigemoto N, Toki S, Takaiwa F (1999). Iron fortification of rice seed by the soybean ferritin gene. Nat Biotechnol.

[CR25] Hassan MJ, Wang F, Ali S, Zhang G (2005). Toxic effect of cadmium on rice as affected by nitrogen fertilizer form. Plant Soil.

[CR26] Horiguchi H, Aoshima K, Oguma E, Sasaki S, Miyamoto K, Hosoi Y, Katoh T, Kayama F (2010). Latest status of cadmium accumulation and its effects on kidneys, bone, and erythropoiesis in inhabitants of the formly cadmium-polluted Jinzu River Basin in Toyama, Japan, after restoration of rice paddies. Int Arch Occ Environ Hea.

[CR27] Inoue H, Higuchi K, Takahashi M, Nakanishi H, Mori S, Nishizawa NK (2003). Three rice nicotianamine synthase genes, *OsNAS1*, *OsNAS2*, and *OsNAS3* are expressed in cells invovled in long-distance transport of iron and differentially regulated by iron. Plant J.

[CR28] Inoue H, Takahashi M, Kobayashi T, Suzuki M, Nakanishi H, Mori S, Nishizawa NK (2008). Identification and localisation of the rice nicotianamine aminotransferase gene *OsNAAT1* expression suggests the site of phytosiderophore synthesis in rice. Plant Mol Biol.

[CR29] Inoue H, Kobayashi T, Nozoye T, Takahashi M, Kakei Y, Suzuki K, Nakazono M, Nakanishi H, Mori S, Nishizawa NK (2009). Rice OsYSL15 is an iron-regulated iron(III)-deoxymugineic acid transporter expressed in the roots and is essential for iron uptake in early growth of the seedlings. J Biol Chem.

[CR30] Ishikawa S, Ae N, Sugiyama M, Murakami M, Arao T (2005). Genotypic variation in shoot cadmium concentration in rice and soybean in soils with different levels of cadmium contamination. Soil Sci Plant Nutr.

[CR31] Ishikawa S, Abe T, Kuramata M, Yamaguchi M, Ando T, Yamamoto T, Yano M (2010). A major quantitative trait locus for increasing cadmium-specific concentration in rice grain is located on the short arm of chromosome 7. J Exp Bot.

[CR32] Ishikawa S, Suzui N, Ito-Tanabata S, Ishii S, Igura M, Abe T, Kuramata M, Kawachi N, Fujimaki S (2011) Real-time imaging and analysis of differences in cadmium dynamics in rice cultivars (*Oryza sativa*) using positron-emitting ^107^Cd tracer. BMC Plant Biol 11:172 10.1186/1471-2229-11-172 PMC3247196 22123026

[CR33] Ishikawa S, Ishimaru Y, Igura M, Kuramata M, Abe T, Senoura T, Hase Y, Arao T, Nishizawa NK, Nakanishi H (2012). Ion-beam irradiation, gene identification, and marker-assisted breeding in the development of low-cadmium rice. Proc Natl Acad Sci U S A.

[CR34] Ishimaru Y, Masuda H, Bashir K, Inoue H, Tsukamoto T, Takahashi M, Nakanishi H, Aoki N, Hirose T, Ohsugi R, Nishizawa NK (2010). Rice metal-nicotianamine transporter, OsYSL2, is required for the long-distance transport of iron and manganese. Plant J.

[CR35] Ishimaru Y, Kakei Y, Shimo H, Bashir K, Sato Y, Uozumi N, Nakanishi H, Nishizawa NK (2011). A rice phenolic efflux transporter is essential for solubilizing precipitated apoplasmic iron in the plant stele. J Biol Chem.

[CR36] Ishimaru Y, Takahashi R, Bashir K, Shimo H, Senoura T, Sugimoto K, OnoK YM, Ishikaw S, Arao T, Nakanishi H, Nishizawa NK (2012). Characterizing the role of rice NRAMP5 in manganese, iron and cadmium transport. Sci Rep.

[CR37] Jalloh MA, Chen J, Zhen F, Zhang G (2009). Effect of different N fertilizer forms on antioxidant capacity and grain yield of rice growing under Cd stress. J Hazard Mater.

[CR38] Johanning J, Strasdeit H (1998). A Coordination-Chemical Basis for the Biological Function of the Phytochelatins. Angew Chem Int Ed.

[CR39] Kakei Y, Ishimaru Y, Kobayashi T, Yamakawa T, Nakanishi H, Nishizawa NK (2012). OsYSL16 plays a role in the allocation of iron. Plant Mol Biol.

[CR40] Khaokaew S, Chaney RL, Landrot G, Ginder-Vogel M, Sparks DL (2011). Speciation and release kinetics of cadmium in an alkline paddy soil under various flooding periods and draining conditions. Environ Sci Technol.

[CR41] Kobayashi T, Nishizawa NK (2012). Iron uptake, translocation and regulation in higher plants. Annu Rev Plant Biol.

[CR42] Kobayashi T, Nakanishi H, Nishizawa NK (2010). Recent insights into iron homestasis and their application in graminaceous crops. Proc Jpn Acad Ser B Phys Biol Sci.

[CR43] Kobayashi T, Itai RN, Nishizawa NK (2014). Iron deficiency responses in rice roots. Rice.

[CR44] Koike S, Inoue H, Mizuno D, Takahashi M, Nakanishi H, Mori S, Nishizawa NK (2004). OsYSL2 is a rice metal-nicotianamine transporter that is regulated by iron and expressed in the phloem. Plant J.

[CR45] Lambert R, Grant C, Sauvé S (2007). Cadmium and zinc in soil solution extracts following the application of phosphate fertilizers. Sci Total Environ.

[CR46] Lang L, Wernitznig S (2011). Sequestration at the cell wall and plasma membrane facilitates zinc tolerance in the moss *Pohlia drummondii*. Envion Exp Bot.

[CR47] Liu X, Jin W, Theil EC (2003). Opening protein pores with chaotropes enhances Fe reduction and chelation of Fe from the ferritin biomineral. Proc Natl Acad Sci U S A.

[CR48] Liu H, Zhang J, Christie P, Zhang F (2008). Influence of iron plague on uptake and accumulation of Cd by rice (*Oryza sativa* L.) seedlings grown in soil. Sci Total Environ.

[CR49] May MJ, Vernoux T, Leaver C, Van Montagu M, Inzé D (1998). Glutathione homeostasis in plants: implications for environmental sensing and plant development. J Exp Bot.

[CR50] Mitchell L, Grant CA, Racz GJ (2000). Effect of nitrogen application of concentration of cadmium and nutrient ions in soil solution and in durum wheat. Can J Soil Sci.

[CR51] Miyadate H, Adachi S, Hiraizumi A, Tezuka K, Nakazawa N, Kawamoto T, Katou K, Kodama I, Sakurai K, Takahashi H, Satoh-Nagasawa N, Watanabe A, Fujimura T, Akagi H (2011). OsHMA3, a P_1B_-type of ATPase affects root to shoot cadmium translocation in rice by mediating efflux into vacuoles. New Phytol.

[CR52] Mori S (1999). Iron acquisition by plants. Curr Opin Plant Biol.

[CR53] Murray-Kolb LE, Takaiwa F, Goto F, Yoshihara T, Theil EC, Beard JL (2002). Transgenic rice is a source of iron for iron-depleted rats. J Nutr.

[CR54] Nakanishi H, Ogawa I, Ishimaru Y, Mori S, Nishizawa NK (2006). Iron deficiency enhances cadmium uptake and translocation mediated by the Fe^2+^ transporters OsIRT1 and OSIRT2 in rice. Soil Sci Plant Nutr.

[CR55] Nocito FF, Lancilli C, Dendena B, Lucchini G, Sacchi GA (2011). Cadmium retention in rice roots is influenced by cadmium availability, chelation and translocation. Plant Cell Environ.

[CR56] Nozoye T, Nagasaka S, Kobayashi T, Takahashi M, Sato Y, Uozumi N, Nakanishi H, Nishizawa NK (2011). Phytosiderophore efflux transporters are crucial for iron acquisition in graminaceous plants. J Biol Chem.

[CR57] Oda K, Otani M, Uraguchi S, Akihiro T, Fujiwara T (2011). Rice *ABCG43* is a Cd inducible and confers Cd tolerance on yeast. Biosci Biotech Biochem.

[CR58] Ogo Y, Kakei Y, Itai RN, Kobayashi T, Nakanishi H, Takahashi H, Nakazono M, Nishizawa NK (2014). Spatial transcriptomes of iron-deficient and cadmium-stressed rice. New Phytol.

[CR59] Olive N, Chadha-Mohanty P, Poletti S, Abrigo E, Atienza G, Torrizo L, Garcia R, Dueñas C, Poncio MA, Balindong J, Manzanilla M, Montecillo F, Zaidem M, Barry G, Hervé P, Shou H, Slamet-Loedin IH (2014). Large-scale production and evaluation of marker-free *indica* rice IR64 expressing phytoferritin genes. Mol Breed.

[CR60] Pich A, Manteuffel R, Hillmer S, Scholz G, Schmidt W (2001). Fe homeostasis in plant cells: does nicotianamine play multiple roles in the regulation of cytoplasmic Fe concentration?. Planta.

[CR61] Rauser WE (1995). Phytochelatins and related peptides. structure, biosynthesis, and function. Plant Physiol.

[CR62] Ravet K, Touraine B, Boucherez J, Briat JF, Gaymard F, Cellier F (2009). Ferritins control interaction between iron homeostasis and oxidate stress in *Arabidopsis*. Plant J.

[CR63] Rehman MZ, Rizwan M, Ghafoor A, Naeem A, Ali S, Sabir M, Qayyum MF (2015). Effect of iorganic amendments for in situ stabilization of cadmium in contaminated soils and its phyto-availability to wheat and rice under rotation. Environ Sci Pollut Res Int.

[CR64] Rizwan M, Ali S, Abbas T, Zia-Ur-Rehman M, Hannan F, Keller C, Al-Wabel MI, Ok YS (2016). Cadmium minimization in wheat: A critical review. Ecotoxicol Environ Saf.

[CR65] Sarwar N, Saifullah MSS, Zia MH, Naeem A, Bibi S, Farid G (2010). Role of mineral nutrition in minimizing cadmium accumulation by plants. J Sci Food Agric.

[CR66] Sasaki A, Yamaji N, Yokosho K, Ma JF (2012). Nramp5 is a major transporter responsible for manganese and cadmium uptake in rice. Plant Cell.

[CR67] Schwab AP, He Y, Banks MK (2005). The influence of organic ligands on the retention of lead in soil. Chemosphere.

[CR68] Shahid M, Dumat C, Khalid S, Niazi NK, Antunes PMC (2016) Cadmium bioavailability, uptake, toxicity and detoxification in soil-plant system. doi:10.1007/398_2016_8 10.1007/398_2016_8 27300014

[CR69] Shao G, Chen M, Wang D, Xu C, Mou R, Cao Z, Zhang X (2008). Using iron fertilizer to control Cd accumulation in rice plants: a new promising technology. Sci China C Life Sci.

[CR70] Sharma SS, Kaul S, Metwally A, Goyal KC, Finkemeier I, Dietz KJ (2004). Cadmium toxicity to barley (*Hordeum vulgare*) as affected by varying Fe nutritional status. Plant Sci.

[CR71] Shimo H, Ishimaru Y, An G, Yamakawa T, Nakanishi H, Nishizawa NK (2011). *Low cadmium (LCD)*, a novel gene related to cadmium tolerance and accumulation in rice. J Exp Bot.

[CR72] Slamet-Loedin IH, Johnson-Beebout SE, Impa S, Tsakirpaloglou N (2015). Enriching rice with Zn and Fe while minimizing Cd risk. Front in Plant Sci.

[CR73] Smilde KW, Van Luit B, Van Driel W (1992). The extraction by soil and absorption by plants of applied zinc and cadmium. Plant Soil.

[CR74] Takahashi M, Terda Y, Nakai I, Nakanishi H, Yoshimura E, Mori S, Nishikawa NK (2003). Role of nicotianamine in the itracellular delivery of metals and plant reproductive devolopment. Plant Cell.

[CR75] Takahashi R, Ishimaru Y, Senoura T, Shimo H, Ishikawa S, Arao T, Nakanishi H, Nishizawa NK (2011). The OsNRAMP1 iron transporter is involved in Cd accumulation in rice. J Exp Bot.

[CR76] Takahashi R, Bashir K, Ishimaru Y, Nishikawa NK, Nakanishi H (2012). The role of heavy-metal ATPases, HMAs, in Zinc and Cadmium transport in rice. Plant Signal Behav.

[CR77] Takahashi R, Ishimaru Y, Shimo H, Ogo Y, Senoura T, Nishizawa NK, Nakanishi H (2012). The OsHMA2 transporter is involved in root-to-shoot translocation of Zn and Cd in rice. Plant Cell Environ.

[CR78] Ueno D, Yamaji N, Kono I, Huang CF, Ando T, Yano M, Ma JF (2010). Gene limiting cadmium accumulation in rice. Proc Natl Acad Sci U S A.

[CR79] Uraguchi S, Fujiwara T (2012). Cadmium transport and tolerance in rice: perspectives for reducing grain cadmium accumulation. Rice.

[CR80] Uraguchi S, Kamiya T, Sakamoto T, Kasaki K, Sato Y, Nagamura Y, Yoshida A, Kyozuka J, Ishikawa S, Fujiwara T (2011). Low-affinity cation transporter (OsLCT1) regulates cadmium transport into rice grains. Proc Natl Acad Sci U S A.

[CR81] Wang H, Wang PF, Zhang H (2009). Use of phosphorus to alleviate stress induced by cadmium and zinc in two submerged macrophytes. Afr J Biotechnol.

[CR82] Wang S, Wang F, Gao S (2015). Foliar application with nano-silicon alleviates Cd toxicity in rice seedlings. Environ Sci Pollut Res Int.

[CR83] Wangstrand H, Eriksso J, Oborn I (2007). Cadmium concentration in winter wheat affected by nitrogen fertilization. Eur J Agron.

[CR84] Weber G, von Wrien N, Hayen H (2008). Investigation of ascoebate-mediated iron release from ferric phytosiderophores in the presence of nicotianamine. Biometals.

[CR85] Wei Y, Shohag MJ, Yang X, Yibin Z (2012). Effects of foliar iron application on iron concentration in polished rice grain and its bioavailability. J Agric Food Chem.

[CR86] White PJ, Broadley MR (2011). Physiological limits to zinc biofortification of edible crops. Front Plant Sci.

[CR87] William LE, Mills RF (2005). P1_B_-ATPases-an ancient family of transition metal pumps with diverse functions in plants. Trends Plant Sci.

[CR88] Wirth J, Poletti S, Aeschlimann B, Yakandawala N, Drosse B, Osorio S, Tohge T, Fernie AR, Günther D, Gruissem W, Sautter C (2009). Rice endosperm iron biofortification by targeted and synergistic action of nicotianamine synthase and ferritin. Plant Biotechnol J.

[CR89] Xie HL, Jiang RF, Zhang FS, McGrath SP, Zhao FJ (2009). Effect of nitrogen form on the rhizosphere dynamics and uptake of cadmium and zinc by the hyperaccumulator *Thlaspi caerulescens*. Plant Soil.

[CR90] Xiong J, An L, Lu H, Zhu C (2009). Exogenous nitric oxide enhances cadmium tolerance of rice by increasing pectin and hemicellulose contents in root cell wall. Planta.

[CR91] Xue Q, Harrison HC (1991). Effect of soil zinc, pH and cultivar uptake in leaf lettuce. (*Lactuca sativa* L. var. *crispa*). Common soil Sci Plant Anal.

[CR92] Yadav R, Arora P, Kumar S, Chaudhury A (2010). Perspectives for genetic engineering of poplars for enhanced phytoremediation abilities. Ecotoxicology.

[CR93] Yamagi N, Xia J, Mitani-Ueno N, Yokosho K, Feng Ma J (2013). Preferential delivery of zinc to developing tissues in rice is mediated by P-type heavy metal ATPase OsHMA2. Plant Physiol.

[CR94] Yamaguchi N, Ishikawa S, Abe T, Baba K, Arao T, Terada Y (2012). Role of the node in controlling traffic of cadmium, zinc, and manganese in rice. J Exp Bot.

[CR95] Yang Y, Chen R, Fu G, Xiong J, Tao L (2016). Phosphate deprivation decreases cadmium (Cd) uptake but enhances sensitivity to Cd by increasing iron (Fe) uptake and inhibiting phytochelatins synthesis in rice (*Oryza sativa*). Acta Physiol Plant.

[CR96] Yang Y, Xiong J, Chen R, Fu G, Chen T, Tao L (2016). Excessive nitrate enhances cadmium (Cd) uptake by up-regulating the expression of *OsIRT1* in rice (*Oryza sativa*). Environ Exp Bot.

[CR97] Yokosho K, Yamaji N, Ueno D, Mitani N, Ma JF (2009). OsFRDL1 is a citrate transporter required for efficient transformation of iron in rice. Plant Physiol.

[CR98] Yoneyama T, Ishikawa S, Fujimaki S (2015). Route and regulation of zinc, cadmium, and iron transport in rice plants (*Oryza sativa* L.) during vegetative growth and grain filling: metal transporters, metal speciation, grain Cd reduction and Zn and Fe biofortification. Int J Mol Sci.

[CR99] Zaccheo P, Crippa L, Pasta VDM (2006). Ammonium nutrition as a strategy for cadmium mobilisation in the rhizosphere of sunflower. Plant Soil.

[CR100] Zhang Y, Xu YH, Yi HY, Gong JM (2012). Vacuolar membrane transporters OsVIT1 and OsVIT2 modulate iron translocation between flag leaves and seeds in rice. Plant J.

[CR101] Zheng L, Cheng Z, Ai C, Jiang X, Bei X, Zheng Y, Glahn RP, Welch RM, Miller DD, Lei XG, Shou H (2010). Nicotianamine, a novel enhancer of rice iron bioavailability to humans. PLoS One.

